# The Role of Exosomal Epigenetic Modifiers in Cell Communication and Fertility of Dairy Cows

**DOI:** 10.3390/ijms21239106

**Published:** 2020-11-30

**Authors:** Pevindu Abeysinghe, Natalie Turner, Isabella Morean Garcia, Eman Mosaad, Hassendrini N. Peiris, Murray D. Mitchell

**Affiliations:** Institute of Health and Biomedical Innovation—Centre for Children’s Health Research, School of Biomedical Sciences, Faculty of Health, Queensland University of Technology, Brisbane, QLD 4029, Australia; abeysinghe.abeysingh@hdr.qut.edu.au (P.A.); natalie.turner@hdr.qut.edu.au (N.T.); isabella.moreangarcia@connect.qut.edu.au (I.M.G.); eman.mosaad@qut.edu.au (E.M.); hassendrini.peiris@qut.edu.au (H.N.P.)

**Keywords:** exosome, epigenetics, miRNA, fertility, inflammation, dairy cow

## Abstract

Abnormal uterine function affects conception rate and embryo development, thereby leading to poor fertility and reproduction failure. Exosomes are a nanosized subclass of extracellular vesicles (EV) that have important functions as intercellular communicators. They contain and carry transferable bioactive substances including micro RNA (miRNA) for target cells. Elements of the cargo can provide epigenetic modifications of the recipient cells and may have crucial roles in mechanisms of reproduction. The dairy industry accounts for a substantial portion of the economy of many agricultural countries. Exosomes can enhance the expression of inflammatory mediators in the endometrium, which contribute to various inflammatory diseases in transition dairy cows. This results in reduced fertility which leads to reduced milk production and increased cow maintenance costs. Thus, gaining a clear knowledge of exosomal epigenetic modifiers is critical to improving the breeding success and profitability of dairy farms. This review provides a brief overview of how exosomal miRNA contributes to inflammatory diseases and hence to poor fertility, particularly in dairy cows.

## 1. Introduction

Poor reproductive efficiency is directly linked with metabolic disorders, a decline in longevity, reduced milk production, greater involuntary culling rate, and higher cow maintenance rates [1]. Dairy cow fertility has declined as a result of selective breeding for elevated milk production without having due consideration for the functional traits responsible for fertility and reproduction [2]. In addition, an activated inflammatory system via immune and/or infectious challenge of the uterus occurring during the postpartum period and higher metabolic pressure due to elevated milk production [3,4,5] can lead to impaired reproduction and fertilization failure [6]. The establishment of successful pregnancies within short calving intervals is a critical factor for cow management systems and the dairy industry [7]. The cumulative effect of these several adverse factors is decreased profitability of the dairy industry [8].

Fertility affects the biomolecules transferred within the body engulfed in exosomes [9,10]. A greater understanding of the role of exosomes as intercellular signaling vesicles in the last decade has resulted in a paradigm shift in our knowledge of how cells communicate [9,10,11,12,13,14,15]. Exosomes can package, carry and deliver their cargo which includes miRNAs [16], messenger RNAs (mRNA) [16], lipids [17] and proteins [18]. Fluorescently labelled bovine milk-derived exosomes given to mice have been observed to travel throughout the body including to the liver, lung, pancreas, kidney, spleen, colon, brain and the ovaries when administered orally or intravenously [19]. Moreover, exosomes are known to be involved in the communications between maternal and fetal tissues which supports the view that exosomes have a role in supporting reproduction/pregnancy [20]. 

The early lactation period has been identified as a time of great metabolic stress for the animal and susceptibility to all forms of the disease occurs during this time [21]. Inflammation is defined as a specific or non-specific immune response to tissue damage or invasion by a foreign body [22,23]. Inflammatory mediators have the potential to deteriorate natural endocrinological pathways and cause fertility disorders [24]. Collectively, inflammatory diseases related to the intrauterine environment of dairy cows and those occurring during the early lactation period negatively influence the reproductive cycle and reduce overall fertility [25,26,27]. 

## 2. Exosomes

Extracellular vesicles were first detected in plasma as procoagulant platelet-derived particles in 1946 [28] and in later years exosomes have been referred to as “platelet dust” [29]. Under the electron microscope, exosomes in solution appear as spheroids but after drying artificially during preparation, canonical exosomes look more biconcave or cup-like in shape [30]. Exosomes are considered as having a size of 30–120 nm [31,32] differentiating them from microvesicles sized from 100–1000 nm [33] with densities of 1.25 g/mL to 1.30 g/mL [34]. Therefore, physically exosomes separate themselves from other vesicular bodies because of their comparatively smaller size and unified shape. 

The bilipid layer of an exosome is rigid [35] and the composition of cholesterol and sphingolipids differ from its parental cell membrane [17]. Exosomes can withstand severe external conditions like low pH, boiling and freezing temperatures [36]. Additionally, exosomes are known to successfully resist the harsh acidic gastrointestinal conditions. Together these factors support the ability and usefulness of exosomes as transport mechanisms in delivering their cargo including miRNA throughout the body and in the circulatory systems of humans and cows [37]. 

### 2.1. Exosome Formation & Cargo Sorting Mechanism

The generation of exosomes begins as an inward budding of late endosomes from the multivesicular body (MVB) membrane in which the endosomes later form into an intraluminal vesicle (ILV) [38] and then release of ILVs into extracellular space as exosomes after fusion with the plasma membrane [39]. Importantly, the incorporation of exosomal cargo and cytosolic elements into pre exosomal bodies happens during the formation of ILVs within MVBs. While some ILVs fuse with plasma membranes to produce exosomes, alternatively the rest of ILVs pass through a degradation pathway within lysosomes [33]. 

There are two suggested mechanisms for the sorting of exosomal cargo into MVBs. One is through an endosomal sorting complex required for transportation (ESCRT) protein machinery which involves four separate proteins ESCRTs (ESCRT-0 to ESCRT-3) [40]. This mechanism cooperates to form MVBs, budding vesicles and sort protein cargo collectively [40,41]. Nonetheless, the exosomal protein “Alix”, has been found in endosomal membrane budding, abscission and exosomal cargo selection processes through syndecan interaction [42]. 

Alternatively, there has been suggested another exosomal cargo sorting pathway which is ESCRT-independent [33]. It involves raft-based microdomains that segregate cargo laterally inside the endosomal membrane [33]. The microdomains are believed to contain sphingomyelinases in high amounts which produce ceramides through hydrolytic removal of phosphocholine moiety from sphingomyelin [43]. 

It has been reported that tetraspanin proteins are also involved in exosome biogenesis in protein loading mechanisms. Ubiquitous specialized membrane platform known as Tetraspanin-enriched microdomain (TEM) involves sorting receptors and signaling proteins into compartments in the plasma membrane [44] and that along with TEM, tetraspanin CD81 is involved in trafficking target receptors in the direction of exosomes [45]. 

### 2.2. Function of Exosomes

Normally gap junctions, cell surface protein/protein interactions, soluble secreted factors like hormones or cytokines are used as cell communicators for signal transduction purposes [33]. Additionally, nucleotides, lipids or short proteins are involved as electrical and chemical signal propagators from cell to cell [46,47]. The ability of exosomes to communicate between cells is evidenced by observations of exosomes from parental cells interacting with the target cells and influencing the behavior and the phenotypic features of the target cells [47]. Exosomes can successfully deliver genetic materials to the target cells through receptor-ligand interactions, the direct fusion of membranes or internalization through endocytosis [48]. Thus exosomes as a mode of intercellular communication [33] are associated with major cellular processes such as immune responses [49], signal transduction [50] and antigen presentation [51]. 

After exosomes enter the target cell, fusion with endosomes enables the horizontal genetic transfer of exosomal cargo to the cytoplasm of the target cell [33]. Bioactive components in exosomal cargo have three pathways (1) connect with surface-bound ligands to stimulate target cells directly (2) transfer their cargo to recipient cells via activated receptors (3) epigenetic reprogramming through miRNA, lipids and functional proteins [52]. 

The functional activity of exosomes differs depending on the originating/parental cell type and the cell’s current fate (i.e., differentiated, stimulated, transformed or stressed), therefore, exosomal cargo also differs [33]. This can then be utilized to gather prognostic information on various diseases [33]. Examples of the use of exosomes in this manner can be seen in cardiovascular and renal diseases [53], lipid metabolic diseases [54], tumor metastasis and angiogenesis [50,55], signal transduction in neurodegenerative disease [56] and chronic inflammation [56]. 

Exosomes also have key regulatory functions in the immune system including antigen presentation [57], immune suppression [58,59,60] and activation [61]. An example of this was described when exosomes were found to drive apoptosis via the antigen-specific pathway for Dendritic Cell (DC) mediated T cell silencing [62]. Furthermore, immune suppression and T-helper cell type 1 (Th1) immune response inhibition has also been reported by regulatory T cell-secreted exosomes which contain Let-7b, Let-7d, and microRNA-155 as exosomal cargo [58]. 

As mentioned earlier, exosomal cargo is a reflection of the physiological status of the secreting tissue. Therefore, the differential expression of exosomal cargo between high- and low-fertility dairy cows is now gaining great attention in the research field [15]. This may lead to novel discoveries that can be the building blocks of new prognostic and/or diagnostic tools to predict dairy cow fertility [15]. 

## 3. Exosomal Epigenetic Modifiers

Exosomes carry complex functional molecules ranging from proteins [18], lipids [17], mRNAs [16] to miRNAs [16]. Exosomes in dairy cows with uterine infections contain more proteins involved in immune system processes than in non-infected dairy cows [32]. Exosomal proteins have been isolated from non-invasive biofluids such as blood plasma [10,14,32], saliva [63,64] and urine [63]. Therefore proteomic content in exosomes is referred to as indicators of disease status [10,65]. Exosomal lipoprotein has been identified as a potential biomarker [66]. Lipidomic analysis has been shown that exosomal phospholipids may have the potential to change the bovine embryonic phospholipid composition in vitro [67]. Exosomal RNA regulate the genome epigenetically, and is thus regarded as the key biomolecule present within an exosome [68]. It has been reported that DNA is not among the exosomal cargo but a considerable amount of RNA has been detected in exosomes isolated from the mast-cell line (MC/9), primary bone marrow-derived mast cells (BMMC) and a human mast-cell line (HMC-1) [69].

In the following sections, we will focus on the epigenetic molecules that can be carried within exosomes.

### 3.1. Exosomal mRNA

Milk exosomal mRNAs are involved in metabolic, degradation and signaling pathways [70,71]. Bovine milk-derived exosomal mRNA affects important physiological and immunological functions on human cells in vitro [72]. A recent report proposes the prediction of clinical stages of bovine leukemia virus (BLV) using mRNA profile from bovine milk EVs [70]. 

### 3.2. Exosomal Non-Coding RNA

Almost 80% of the transcribed genes of the mammalian genome are non-coding [73,74]. Non-coding RNAs can be categorized into long non-coding RNAs (lncRNA; >200 nucleotides) [75] and miRNA (19–24 nucleotides) [74]. Non-coding RNA regulates the expression level of their target DNA, RNA or protein molecules through various mechanisms such as epigenetic, transcription, post-transcriptional regulations [76]. Stress or disease status triggers aberrant expression of non-coding RNA and that allows the use of non-coding RNAs as specific biomarkers to predict various pathologies [77]. 

#### 3.2.1. Exosomal Long Non-Coding RNA

A very small number of studies cover research on exosomal lncRNA related to fertility. But a recent study on bovine milk exosome-derived lncRNA has been reported a profile of lncRNAs involve in immunity, development and reproduction [78]. Nonetheless, exosomal lncRNA shows stability in the human digestive system and in harsh environmental conditions in vitro [78]. However, so far miRNA is so far regarded as the main regulatory molecule within exosomal cargo [68].

#### 3.2.2. Exosomal miRNA 

Exosomal miRNA plays a vital role in inter-cellular and inter-organism level signal transduction. MiRNAs are theorized to act as an epigenetic regulators acting on approximately 60% of all mammalian genomes. [74,79,80]. They are complementary to the mRNAs 3’ untranslated region (3’ UTR) of its target [79]. Their interactions lead to the inhibition of translation and less frequently leads to mRNA degradation [79]. The miRNAs found in the exosomes are reported to have multiple functions; for example, mir-1 is involved in cardiomyocyte differentiation and proliferation [81], miR-17 is upregulated in B-cell lymphoma [82], miR-181 is involved in hematopoietic cell differentiation [83] and miR-375 is responsible for insulin secretion [84]. Together these findings suggest the extensive regulatory capacity of miRNAs when coupled to the intercellular communication abilities of an exosome [69].

Bovine milk contains high amounts of exosomes and the exosomal cargo contents include biologically active molecules [85,86]. The miRNA content from cow milk-derived EVs shows a great diversity [86]. Therefore, milk exosomal contents can utilize as prognostic biomarkers for many infectious diseases [70]. A considerable number of exosomal miRNAs are contain in bovine milk with great stability [85]. This is evidenced by observation of a non-affected total RNA yield after the acidification of bovine milk [87] and microwave heating only depleting 40% of miRNA-29b but not affecting the yield of miRNA-200c [88]. Ultrasonication of bovine colostrum exosomes resulted in the inhibition of immune regulating function suggesting that the integrity of the exosomal membrane is vital for modulating exosomal miRNA function [89]. Moreover, miRNA are reported to be stable with effective retrieval and analysis of miRNAs possible from formalin-fixed paraffin-embedded tissues [90,91]. Other processing practices of bovine skim milk resulted in variations in miRNA quantity e.g., pasteurization and homogenization were also observed to result in a dramatic loss of miRNA [88] and fermentation resulting in alterations to exosome and miRNA number [92]. An additional benefit of milk exosomes is its ability to be tolerated across species [19]. Bovine milk exosomes loaded with chemotherapeutic and chemopreventive agents have shown higher efficacy against lung tumor xenografts in vivo [19]. Thus, there is potential to utilize these milk exosomes as a vehicle for drug delivery [19]. 

**Figure 1:**
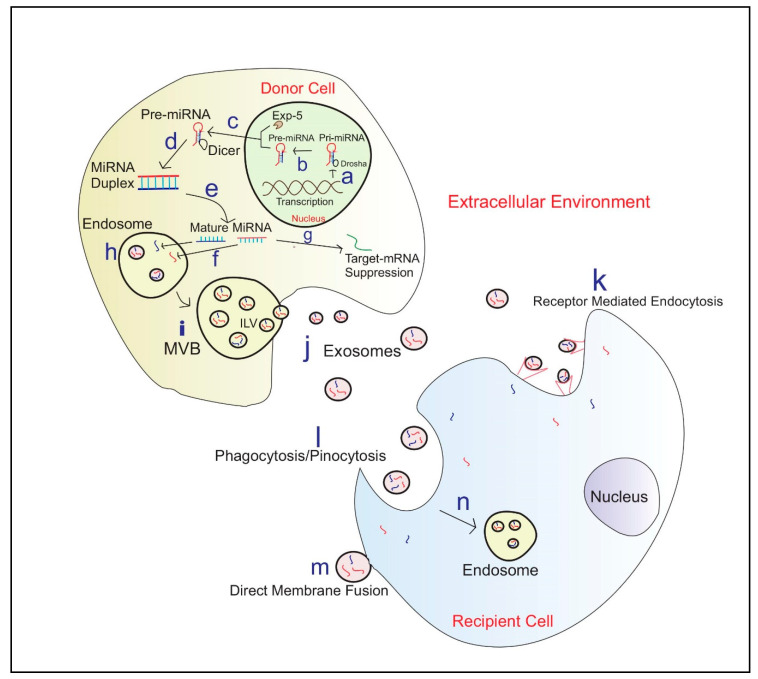
Exosome biosynthesis and miRNA Communication between cells **a**—Translation of primary miRNA (Pri-miRNA) from DNA; **b**—Cleavage of pri-miRNA to precursor miRNA(Pre-miRNA) by microprocessor complex including Drosha; **c**—Exportation of Pre-miRNA to the cytoplasm by Exportin 5(EXP-5); **d**—Pre-miRNA processing into miRNA duplexes using Dicer; **e**—Duplex unwinds to produce mature miRNA; **f**—miRNA engulf into endosomes; **g**—Few miRNA’s contribute to target mRNA silencing in the Donor cell; **h**—Endosomes engulfed miRNA; **i**—Micro-vesicular Bodies (MVB) release Intraluminal Vesicles (ILV) fuse with the plasma membrane **j**—Exosomes carrying miRNAs; **k**—Exosome entry to recipient cell via receptor-mediated endocytosis; **l**—Entrance of exosomes via Phagocytosis/Pinocytosis; **m**—Exosome entry via Direct membrane fusion; **n**—Formation of Endosome in the recipient cell and then exosomal miRNA release into the cytoplasm.

Exosomal miRNAs show a higher resistance to adverse external conditions [85]. This suggests exosomes may play a key role in the horizontal transfer of miRNA by protecting the miRNAs within their plasma membrane [85,93]. The biosynthesis of exosomal miRNA packaging and communication between cells is illustrated in Figure 1. 

#### Circulating miRNA vs. Exosomal miRNA

It has been reported that the miRNAs present within exosomes were different from the miRNA content of the cell which infers that certain miRNAs are packaged into the exosomes in a unique selective way [69]. A circulating miRNA array profiling serum revealed differentially regulated miRNAs opening up the possibility of these miRNAs as potential therapeutic biomarkers easily accessible from non-invasive, readily available body fluids [94]. In fact, it has been shown that miR-17-5p, miR-20a and miR-22 are downregulated in plasma samples from patients with endometriosis [95]. We have shown that bovine endometrial cells decreased their production of prostaglandin E_2_ (PGE_2_) following treatment with exosomes loaded with miRNA-143 (which is a known inhibitor of cyclooxygenase-2 (COX-2), an enzyme required for the production of PGE_2_) [96]. This effect was reversed by an antagonist of miRNA-143 [96]. The association of miRNAs in inflammation-related diseases has also been reported. Mir-483-5p competitively binds to the 3’ UTR of mRNA Insulin-like growth factor 2 (IGF2) in ovarian endometriosis patients which results in the overgrowth of the endometrial tissue outside the uterus [97]. In a cohort of ovarian endometriosis patients’, 107 miRNAs and 6112 mRNAs were identified [98]. These studies provide support for the use of miRNAs from easily accessible, non-invasive body fluids such as milk, saliva, blood plasma as diagnostic biomarkers and/or the potential to develop/target them into therapeutics which can be used to treat health disorders.

## 4. Exosomal Epigenetic Modifiers and Fertility

**Figure 2:**
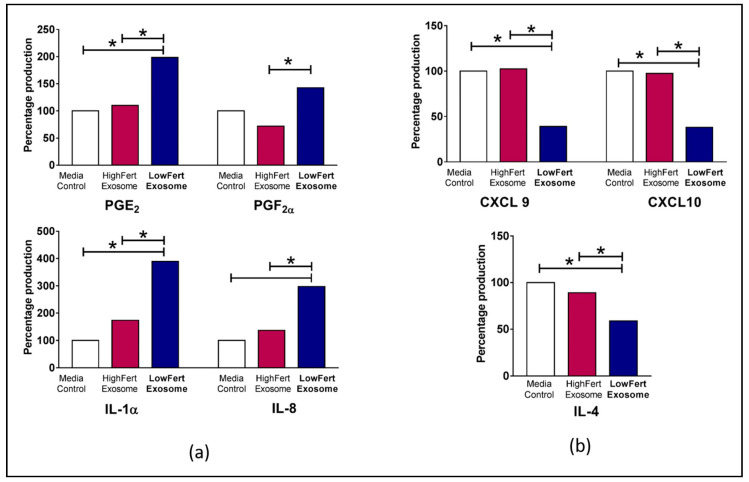
Differential expression of cytokines in bovine endometrial cells (**a**) Increased relative fold expression of prostaglandin E_2_ (PGE_2_), prostaglandin F_2_ alpha (PGF_2α_), interleukin 1 alpha (IL1α), (C-X-C motif) ligand 8/interleukin-8 (CXCL8/IL8) in low fertile exosome loaded cells. (**b**) Decreased relative fold expression of (C-X-C motif) ligand 9 (CXCL9), (C-X-C motif) ligand 10 (CXCL10) and interleukin 4 (IL-4). Differences of *P* < 0.05 are considered statistically significant between sample groups denoted by *. Adapted by experimental data published in Koh et al., 2020 [9].

Biological factors such as mRNA/protein abundance and turnover can influence the differential expression of inflammatory mediator genes [99,100]. Cytokine mRNAs including interleukin-8 (IL-8), interleukin-1-beta (IL-1β) have been expressed differentially and influence cytokine response in cervicovaginal epithelial cells [101]. A recent study shows that circulating exosomes from low fertile groups alter the expression of inflammatory mediators responsible for aberrant endometrial inflammation in bovine endometrial epithelial (bEEL) and stromal (bCSC) cells [9]. Figure 2 shows elevated expression of interleukin 1 alpha (IL1α), (C-X-C motif) ligand 8/interleukin -8 (CXCL8/IL8, prostaglandin E_2_ (PGE_2_) and prostaglandin F_2_ alpha (PGF_2α_) in cells with low fertility exosomes [9]. Increased chemokine and cytokine production results in poor endometrial function which in turn negatively affects fertility and pregnancy success [9,102].

**Figure 3:**
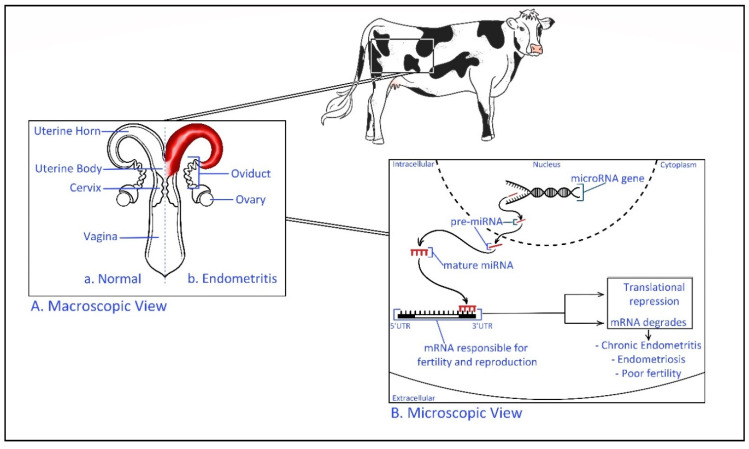
Epigenetic regulation of bovine uterine inflammatory diseases. (**A**). Macroscopic view—Comparison of a healthy and endometritis infected bovine reproductive system Endometritis is defined as a localized infection of the uterus lining [107]. (**B**). Microscopic view—Transcribed miRNA precursor (pre-miRNA) transfer to the cellular cytoplasm before mature miRNA targets the 3′ untranslated region (UTR) of specific mRNA. The inactive complex of (miRNA-mRNA) repress the translation process and the fate of mRNA is to be degraded. This may result in inflammatory diseases such as endometritis and poor fertility. For example, miR-218 has shown to be involved in the pathogenesis of bovine endometritis by inhibiting the activating mRNA of macrophage inflammatory protein (Mip-1) chemokine [108].

The relationships between miRNA and fertility have been revealed recently exposing the severity of the epigenetic behavior of miRNA in exosomal cargo [103]. Higher expression of miR-145 has been identified in recurrent implantation failure (RIF) patients [103]. This miRNA is suggested to target the sequence of mRNA coding the insulin-like growth factor 1 receptor (IGF1R) gene, which is a gene activated in the adhesion of the embryo to endometrial tissue [103]. It has been found that in a mouse model, miR-199a represses the expression of integral transmembrane mucin glycopeptide Mucin 1, which plays a key role in embryo attachment enabling successful implantation [104]. In the cow exosome miRNA derived from uterine fluids of dairy cows with uterine inflammatory diseases has shown a differentially expressed miRNAs than the healthy controls, and, the report has predicted that exosomal miRNA may affect the fertility within the herd [105]. Therefore, exosomal miRNAs have a potential role as an epigenetic regulator of biological signaling pathways of the reproductive cycle during pregnancy (Figure 3) [106]. 

### DNA Methylation and Histone Acetylation

DNA modifications in the exosomal cargo and the consequent epigenetic modification are responsible for tumor progression [109,110,111]. Tumor regulating exosomal miR-652-5p has shown a high level of upstream DNA hypermethylation in tumor tissues and serum samples of oesophageal squamous cell carcinoma (OSCC) [109]. DNA methylation BarH-like 2 homeobox protein (BARHL2) in exosomal DNA from gastric juice has been recently established as a biomarker to predict gastric cancer [112]. Hypermethylation in the tumor suppressor genes P53 and RIZ1 were observed in cells co-incubated with leukemia-derived microvesicles [113]. Level of promotor methylation to mRNA of O6-methylguanine DNA methyltransferase (MGMT) changed during drug treatment to glioblastoma patients [114]. Thus, exosomes transmit information involved in methylation to the recipient cells, ultimately inducing tumor-related microenvironments [111].

Apart from exosomal cargo, modifications to the structure of the DNA and histone proteins regulate fertility [115,116]. Genes with conserved non-methylated promoters are involved in embryo development in bovine species [117]. Recently, the degree of methylation in the promoter region of Bovine *Vasa* Homology (*Bvh*) gene has been reported to be higher in dairy bulls with poor sperm motility [118]. It has been shown that DNA hypermethylation disrupts P-element induced wimpy testis (PIWI)-interacting RNA (piRNAs) production which causes hybrid male sterility (HMS) [119]. The cytosine-guanine dinucleotides islands (CpG island) have been reported differentially methylated between high and low motile *Bos taurus* sperm populations [120]. DNA methylation has been induced as a result of exposure to toxins. For example, chlorpyrifos (CPF) an organophosphate pesticide, alters sporadic methylation levels in male gametes in vitro [116]. Similar DNA methylation patterns have been shown between bovine blastocytes and gametes from various in vitro conditions in a recent study to demonstrate parental effects on epigenetic programming [121]. Identification of methylation signatures in non-small cell lung cancer (NSCLC) showcases the potential of DNA methylation as a prognostic tool [122].

Bioinformatic analysis has shown histone acetylation gene overlap between exosomal mRNA and proteins in transgenerational epigenetic inheritance [123]. An oligodendroglioma cell line has released extracellular vesicles with differentiation-specific linker histone H1˚ [124] which was associated with terminal differentiation [125].

Non-exosomal epigenetic modification (especially acetylation) in histone affects embryo implantation and spermatogenesis [126,127]. It has been demonstrated that the expression of sperm hyperacetylated histone 4 (H4) was reduced in low fertility bulls which results in loose chromatin structures [126]. Abnormal acetylation and methylation in histone three lysine 27 (H3K27) in bovine spermatozoa disrupt activation after fertilization [128]. Metabolic disturbances such as ketone body β-hydroxybutyrate (BOHB) has been demonstrated as an inhibitor of H3K27 and hyperacetylation [129]. Histone deacetylases (NIHDAC) play an important role in female fertility by regulating ovary maturation or ovipositor development [130]. Loss of histone acetylation inhibits chromatin accessibility and nucleosome eviction during spermiogenesis which leads to poor fertility in males [131]. Exposure to toxins such as arsenite has been reported to induce ubiquitination and acetylation of histone which associate with male infertility [132]. Histone acetylation and DNA methylation caused by addictive stimulants (for example, cocaine) have resulted in poor reproductive function in male mice [133]. Nonetheless, post-translational modifications are significant for the fertility and function of seminal proteins in mature sperm [134]. Histone modifications can be utilized to distinguish between aberrant clinical conditions [135].

## 5. Conclusions 

Inflammation in the reproduction tract alters the natural reproductive cycle and ultimately diminishes the potential fertility and pregnancy success of a dairy cow. Additionally, dairy cows suffer from common postpartum uterine inflammatory diseases, e.g., mastitis, metritis and endometritis. These diseases affect dairy cow management by requiring additional labor and financial resources to maintain, treat or cull animals. Exosomes offer a potential method for the evaluation and diagnosis of inflammatory, reproductive and fertility disorders through exosomal content characterization and comparison. Moreover, the ability of exosomes to transport biological molecules including miRNA (i.e., critical epigenetic regulator) could be manipulated as a method for the delivery of therapeutic agents to alter cellular pathways for the benefit of a dairy cow’s reproductive health. So far, successful isolation techniques of miRNA from exosomes are being explored. However, the diagnostic and therapeutic potential of exosomal miRNA to improve bovine fertility is yet to be explored fully.

## Abbreviations

BARHL2	BarH-like 2 homeobox protein
bCSC	Bovine endometrial stromal
bEEL	Bovine endometrial epithelial
BLV	Bovine leukemia virus
BOHB	Body β-hydroxybutyrate
Bvh	Bovine *Vasa* Homology
BMMC	Bone marrow-derived mast cells
CD4+	Cluster of differentiation—4
CD81	Cluster of differentiation—81
COX-2	Cyclooxygenase-2
CPF	Chlorpyrifos
CpG island	Cytosine-guanine dinucleotides islands
CXCL8/IL8	C-X-C chemokine ligand 8/Interleukin 8
CXCL9	(C-X-C motif) ligand 9
CXCL10	(C-X-C motif) ligand 10
DC	Dendritic cells
ESCRT	Endosomal sorting complex required for transportation
EV	Extraccellular vesicles
H3K27	Histone three lysine 27
H4	Hyperacetylated histone 4
HMC-1	human mast-cell line
IGF2	Insulin-like growth factor 2
IGF1R	Insulin like growth factor 1 receptor
IL1α	Interleukin 1—alpha
IL-4	Interleukin—4
ILV	Intraluminal Vesicles
Let-7b	Lethal—7b
Let-7d	Lethal—7d
LH	Luteinizing hormone
lncRNA	Long non-coding RNA
MC/9	Mast-cell line/9
MGMT	O6-methylguanine DNA methyltransferase
MIP-1	Macrophage inflammatory protein
miRNA	Micro RNA
mRNA	Messenger RNA
MVB	Micro-vesicular Bodies
NIHDAC	Histone deacetylases
NSCLC	Non-small cell lung cancer
PGE_2_	Prostaglandin E_2_
PGF_2α_	Prostaglandin F_2_ alpha
piRNAs	P-element induced wimpy testis (PIWI)-interacting RNA
pre-miRNA	Precursor micro RNA
pri-miRNA	Primary micro RNA
RIF	Recurrent implantation failure
TEM	Tetraspanin-enriched microdomain
Th1	T—helper cell type 1
UTR	Untranslated region
